# Data-driven acceleration of mixed-integer bilinear programs: a comparative study for robot motion planning

**DOI:** 10.3389/frobt.2025.1656564

**Published:** 2026-01-08

**Authors:** Xuan Lin

**Affiliations:** 1 Robotics and Mechanisms Laboratory, Department of Mechanical and Aerospace Engineering, University of California, Los Angeles, CA, United States; 2 The Laboratory for Intelligent Decision and Autonomous Robots, George W. Woodruf School of Mechanical Engineering, Georgia Institute of Technology, Atlanta, GA, United States

**Keywords:** trajectory optimization, robot motion planning, optimal control, mixed-integer bilinear programs, mathematical programs with complementarity constraints

## Abstract

This paper presents a comparative study of data-driven acceleration techniques for mixed-integer bilinear programs (MIBLPs) applied to robot motion planning. MIBLPs combine discrete decision variables and nonlinear constraints, making them computationally challenging for real-time robotics applications. We investigate two reformulation strategies: (1) converting binary variables into continuous variables with complementarity constraints (MPCC), and (2) converting bilinear constraints into mixed-integer linear constraints using McCormick envelopes (MICP). Using offline computed solutions as datasets, we apply K-nearest neighbor methods to warm-start both reformulations. We experimented with the proposed data-driven MIBLP formulation for motion planning on a linear inverted pendulum with contacts, and planning motion using a single rigid body model with mode transitions and contacts. Our results demonstrate that when sufficient data is available, MICP achieves consistently fast solving speeds that are suitable for real-time computation, while MPCC achieves higher success rates with limited amount of data. Our approach is capable of planning motions for the SCALER robot platform to transition between bipedal and quadrupedal configurations to navigate around obstacles without pre-specified gaits. Code for reproducing our results is available at https://github.com/XuanLin/MIBLP_benchmark.

## 1 Introduction

Optimization-based methods are powerful tools for solving robotic motion planning problems. For problems involving discrete decisions and nonlinear constraints, typical approaches include mixed-integer convex programs (MICPs) (Deits and Tedrake, 2014; Lin et al., 2019), which explicitly handle discrete variables using techniques like Branch and Bound (Boyd and Mattingley, 2007) or Benders Decomposition (Geoffrion, 1972; Lin, 2024), and nonlinear programs (NLPs) (Dai et al., 2014; Winkler et al., 2018; Shirai et al., 2020), which deal with nonconvex constraints. Each approach has distinct advantages and limitations when solved with off-the-shelf solvers. MICP solvers can find global optimal solutions without any initial guess but often require prohibitively long solving times for problems with numerous integer variables. NLPs, while computationally more efficient when solved with e.g., interior point methods (Wachter, 2002), tend to converge to local optimal solutions that may exhibit inconsistent behavior sensitive to initial guesses. To introduce discrete variables, NLPs require complementary constraints (Yunt and Glocker, 2006; Posa et al., 2014), which is numerically challenging as it violates the majority of Constraint Qualifications established for standard nonlinear optimization (Luo et al., 1996). Combining MICPs and NLPs leads to mixed-integer nonlinear programs (MINLPs), such as mixed-integer bilinear programs (MIBLPs), which offer greater descriptive power for formulating complex problems but become computationally intractable unless the problem size is very small (Marcucci, 2024). This limits the practical implementation of MINLPs in online applications.

Researchers have been investigating machine learning methods to accelerate optimization solving procedures. Data can be gathered to enhance both general-purpose solvers and problem-specific approaches. For example, MIP solvers such as Gurobi (Gurobi Optimization, LLC, 2024) use branch-and-bound algorithms that heavily rely on cutting planes and heuristics to discover better solutions and eliminate infeasible ones. These techniques used inside solvers can be aided by machine learning methods (Nair et al., 2020; Tang et al., 2020). While these approaches aim to improve general solver performance, they require modifying solver internals and typically demand large-scale training datasets spanning many problem classes. Additionally, recent work has also explored neural network-based warm-starting for trajectory optimization in robotics, such as using diffusion models (Carvalho et al., 2023; Li et al., 2024), constraint-informed learning (Briden et al., 2025), and applications including navigating challenging obstacles in unfamiliar environments (Dalal et al., 2024) and trajectory generation on the International Space Station (Banerjee et al., 2025).

On the other hand, for parametric optimization problems where parameters follow specific distributions, solved problem instances can be collected offline to learn problem-solution mappings that accelerate online solving. In this problem-specific setting, simpler learning schemes such as K-nearest neighbors can be effective. This approach has been investigated for MICPs (Zhu and Martius, 2020; Cauligi et al., 2021), and NLPs (Hauser, 2016). These data-driven methods have demonstrated potential in reducing computation time for complex optimization problems. However, considerably less work has been done on applying these data-driven techniques to mixed-integer nonlinear programs (MINLPs) (Dua and Pistikopoulos, 1999), particularly in comparing different reformulation strategies.

In this paper, we investigate data-driven techniques for accelerating mixed-integer bilinear programs (MIBLPs), a common class of MINLPs, for robotics applications. Rather than developing an entirely new approach, we examine how to reformulate MIBLPs into alternative, more tractable formulations such as MICPs or NLPs, then apply simple-yet-effective KNN-based data-driven methods. We systematically compare the performance of these reformulations in terms of solving speed, success rate, solution quality, and data requirements—a comparative perspective that has received limited attention in prior works.

Several established approaches to reformulate MIBLPs exists in the literature. In this paper, we investigate two widely-accepted formulations:

1. Turning binary variables into continuous variables with complementarity constraints, transforming the problem into a mathematical program with complementarity constraints (MPCC) (Park and Boyd, 2018). This formulation has been used in previous works such as (Posa et al., 2014). This formulation enables the direct application of data-driven methods such as (Hauser, 2016; Lin et al., 2022).
2. Converting bilinear constraints into mixed-integer linear constraints using McCormick envelopes (Gupte et al., 2013), which transforms the problem into an MICP. This formulation has been used in previous work such as (Dai et al., 2019). This formulation enables the direct application of data-driven methods such as (Zhu and Martius, 2020; Cauligi et al., 2021).

Given these reformulation options, we ask a natural question: *Given a certain amount of problems solved offline as warm-start data, which re-formulation can solve the original MIBLP faster and more reliably? How much data is needed to achieve such performances?*

**FIGURE 1:**
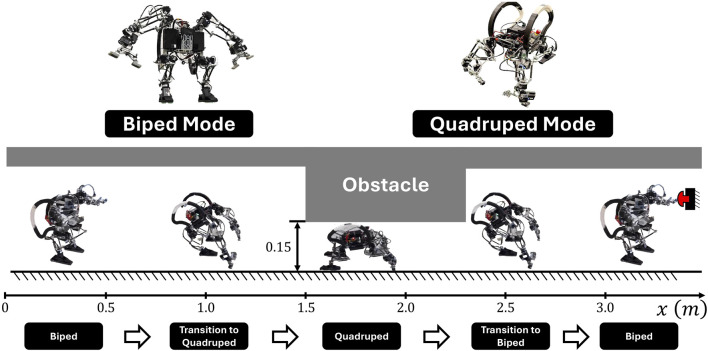
Experimental demonstration: SCALER robot transitions from bipedal to quadrupedal form to navigate beneath an obstacle, then returns to bipedal form to reach an elevated target position (illustrated by a button-pushing task).

To answer these questions, we conduct a comparison of different MIBLP reformulations in the context of robotic motion planning and control. We experiment with motion planning problems formulated as MINLPs on two models: a linear inverted pendulum with soft contact walls, and a single rigid body with contact. The inverted pendulum problem allows us to study the performance of reformulations in a lower-dimensional setting, while the single rigid body control problem with contact represents a more complex scenario commonly seen in legged locomotion (Ding et al., 2022) and manipulation (Hogan and Rodriguez, 2020) tasks. In this paper, we use the single rigid body model to study a locomotion problem where the robot must change its mobility mode from biped to quadruped to overcome an obstacle, as illustrated in Figure 1. For each problem, we apply data-driven methods to both the MPCC and MICP reformulations, comparing their success rate, computational speed, and solution quality as the amount of training data increases.

To summarize, our contributions are as follows:

1. Formulate two robotics control problems (inverted pendulum and single rigid body with mode transitions and contacts) as MIBLPs and solve them with data-driven methods.
2. Benchmark the data-driven performance of different MIBLP re-formulations across these robotic problems.

A preliminary version of this work was published (Lin et al., 2024). While the conference paper introduced the comparative study of MICP and NLP reformulations for MIBLPs, it was limited to the simplified Book Placement Planning problem without dynamics. The current paper significantly extends this work by evaluating the reformulation strategies on more challenging robotic systems with complex dynamics, including an inverted pendulum and single rigid body with contacts.

## 2 Related works

### 2.1 Parametric programming

The field of parametric programming focuses on constructing mappings from optimization problem parameters to their corresponding solutions (Fiacco, 1983; Pistikopoulos et al., 2002). This approach has been explored across various optimization domains, including linear programming (Gal, 2010), quadratic programming (Bemporad et al., 2002), and mixed-integer nonlinear programming (Dua and Pistikopoulos, 1999). A notable application of parametric programming appears in controller design through explicit Model Predictive Control (MPC) (Bemporad et al., 2002; Tøndel et al., 2003), which involves solving MPC problems offline and storing the resulting active constraint sets. During online computation, the current system parameters are used to identify the appropriate pre-computed solution. Bemporad et al. (2002) demonstrated that for constrained linear quadratic regulator problems, these active sets form polyhedrons. Dua and Pistikopoulos (2000) further proposed efficient parameter space partitioning algorithms to construct those polyhedrons.

For non-convex optimization problems, where active sets generally do not form polyhedrons, researchers have developed different approaches. Several studies (Hauser, 2016; Tang and Hauser, 2019; Zhu and Martius, 2020) employ non-parametric learning techniques such as K-nearest neighbor (KNN) to directly leverage stored solution data for generating warm-start solutions online. A more recent research has expanded these methods by incorporating neural networks to learn embeddings that map from larger parameter sets to optimization solutions (Cauligi et al., 2021). A significant advantage of neural network methods is their capability to generalize to out-of-distribution problem instances that lie considerably outside the training dataset (Power and Berenson, 2022).

### 2.2 Mixed-integer convex programs

Mixed-integer convex programs (MICPs) integrate both continuous and discrete decision variables within an optimization framework. Their formal mathematical definition hinges on the property that if all binary variables are relaxed (i.e., replaced by continuous variables between 0 and 1), the resulting problem must be convex (Conforti et al., 2014). Standard MICP solvers such as Gurobi leverage branch-and-bound methods (Boyd and Mattingley, 2007) and cutting plane techniques (Marchand et al., 2002). Although MICPs face exponential worst-case computational complexity with respect to the number of binary variables, practical experience reveals that many real-world problems can be solved by exploring only a small portion of the potential search tree (Williams, 2013). This has facilitated the deployment of mixed-integer approaches in time-sensitive robotics applications such as online motion planning (Tordesillas et al., 2019). Nevertheless, as problem complexity increases, solution times can still extend to minutes or even hours (Lin et al., 2021), presenting significant challenges for real-time implementation.

### 2.3 Mathematical programming with complementary constraints

Mathematical programs with complementarity constraints (MPCCs) offer an alternative approach to handling discrete variables by reformulating them as continuous variables with additional complementarity conditions. These complementarity constraints enforce that when one variable takes a non-zero value, the other must be exactly zero. This formulation, while compact, presents significant computational challenges because it violates the majority of Constraint Qualifications established for standard nonlinear optimization (Luo et al., 1996). To address these difficulties, researchers have developed specialized algorithms including time-stepping methods (Anitescu and Potra, 1997), pivoting algorithms (Drumwright, 2015), and central path approaches (Kojima et al., 1991). In robotics applications, complementarity constraints have proven valuable for trajectory optimization across different locomotion gaits and implicit contact dynamics (Yunt and Glocker, 2006; Posa et al., 2014; Zhang et al., 2021; Le Cleac’h et al., 2024), where they capture the complementary relationship between contact forces and surface distances. Complementarity constraints can also be used to model binary decision variables (Park and Boyd, 2018).

## 3 Materials and methods

### 3.1 Data-driven parametric MIBLPs

Consider a set of optimization problems parameterized by $\Theta \in R^{p}$ , drawn from a distribution D(Θ) . We aim to solve a parameterized mixed-integer bilinear program (MIBLP) (Fischetti and Monaci, 2020):

$$\underset{x,\;z}{\text{minimize}}\|x-x_{g}\left(\Theta \right){\|}_{Q}^{2}$$ (1a)

$$\text{s.t.}\;\;A\left(\Theta \right)x\leq h\left(\Theta \right)$$ (1b)

$$l_{i}\leq x\left[i\right]\leq u_{i}\;\;\;i=1,\ldots ,\text{dim}\left(x\right)$$ (1c)

$$x\left[j\right]\in \left\{0,1\right\}\;\;\;\;j\in J$$ (1d)

$$x\left[r_{s}\right]=x\left[p_{s}\right]x\left[q_{s}\right]\;s=1,\ldots ,S$$ (1e)

where $x\in R^{\text{dim}\left(x\right)}$ includes both continuous and binary variables, $x_{g}$ denotes the desired target values as a function of the parameters, and J specifies the index set for binary variables in x . Constraints (1b) are mixed-integer linear, meaning that if the binary variables are relaxed to continuous domains (i.e., replacing x[j]∈{0,1} with x[j]∈[0,1],∀j∈J ), these constraints become linear. Note that equality constraints can be incorporated into (1b) by expressing them as pairs of opposing inequality constraints. Constraints (1c) provide simple bounds on the variables, while (1d) enforces the binary requirements. Finally, (1e) introduces bilinear constraints, the distinguishing feature of MIBLPs compared to mixed-integer linear or quadratic programs.

As introduced in Section 1, we compare the data-driven performance of two reformulation approaches: converting problem (1) into either an MICP or an MPCC, and solving these reformulations with data-driven methods. For the MICP reformulation, when a learning algorithm can predict binary variable assignments based on problem parameters Θ , the original problem (1) reduces to a convex optimization problem that can be efficiently solved with standard convex program solvers. Alternatively, for the MPCC reformulation (leading to an NLP with complementary constraints), when a learning algorithm provides high-quality initial guesses for all variables, it can significantly improve computational performance.

To implement these data-driven approaches, we first construct a dataset of size D consisting of tuples $\left\{\left(\Theta_{i},x_{i}\right)\right\}$ for i=1,…,D . The parameters $\left\{\Theta_{i}\right\}$ are drawn from D(Θ) , and each corresponding solution $x_{i}$ is obtained by solving the original problem using conventional non-data-driven methods. This offline computation, while potentially time-consuming, needs to be performed only once.

We then use this dataset to train models that can either predict binary variables for the MICP reformulation, or generate initial guesses for all variables for the MPCC reformulation. The MICP approach only requires binary variable prediction since fixing these variables yields a convex programming problem that can be solved efficiently without warm-starting. Formally, we seek to approximate the problem-solution map F: Θ↦x using the collected data. For binary variable prediction, we focus on learning $F_{b}:\;\Theta \mapsto \left\{x\left[j\right]|j\in J\right\}$ , while for initial guess generation, we approximate the full mapping F . In the following sections, we describe the implementation details for each reformulation approach.

#### 3.1.1 MPCC Re-formulation

One approach to reformulating problem (1) involves converting all binary variables into continuous variables with complementarity constraints, transforming (1) into MPCCs that can be solved using NLP solvers. In this reformulation, we remove constraint (1d) and instead impose the equivalent complementarity constraint proposed by Park and Boyd (2018):

$$x\left[j\right]\left(1-x\left[j\right]\right)\leq \epsilon \;\forall j\in J$$ (2)

where ϵ>0 is a small constant that slightly relaxes the constraint to improve numerical convergence properties. We have also experimented with alternative complementarity formulations from Stein et al. (2004) but observed similar or worse computational performance.

Using our constructed dataset, we implement the approach proposed by Hauser (2016) which utilizes a K-nearest-neighbor (KNN) algorithm to generate multiple candidate initial guesses $x_{0}$ for a given online problem with parameters Θ . Specifically, for each new problem instance, we identify the K most similar problem instances in our dataset based on Euclidean distances in the parameter space and use their solutions as initial guesses. These initial guesses are used for solving the MPCC one by one until a feasible solution is found or all candidates are exhausted. This complete algorithm is presented in Algorithm 1.

Algorithm 1 MPCC Online Solving Procedure.

Input: $\left\{\left(\Theta_{i},x_{i}\right)\right\}$ , Θ

1:  Find the K-nearest-neighbors $\Theta_{1}$ , …, $\Theta_{K}$ in $\left\{\Theta_{i}\right\}$ , sorted in order of increasing Euclidean distance $d\left(\Theta ,\Theta_{k}\right)$ . Retrieve $x_{1}$ , …, $x_{K}$ .
2: for k=1,…,K do
3:  Solve the MPCC re-formulation of (1) that replaces constraint (1d) with constraint (2) using applicable NLP solvers with $x_{k}$ as initial guess.
4:  if *successful* then
5:    return x

6: return *nil*

#### 3.1.2 MICP Re-formulation

Another reformulation approach converts bilinear constraints into mixed-integer linear constraints. The key idea is to partition the ranges of the variables in each constraint (1e) into small intervals, then approximate the constraint within each interval using the linear McCormick envelope relaxation (McCormick, 1976). For the bilinear constraint $x\left[r_{s}\right]=x\left[p_{s}\right]x\left[q_{s}\right]$ , we divide the range of $x\left[p_{s}\right]$ into $n_{p_{s}}$ intervals with breakpoints $\left[l_{p_{s}},x_{p_{s}}^{1},\ldots ,x_{p_{s}}^{n_{p_{s}}-1},u_{p_{s}}\right]$ , and similarly divide $x\left[q_{s}\right]$ into $n_{q_{s}}$ intervals with breakpoints $\left[l_{q_{s}},x_{q_{s}}^{1},\ldots ,x_{q_{s}}^{n_{q_{s}}-1},u_{q_{s}}\right]$ .

We then use the Special Ordered Set of type 2 (SOS2) formulation introduced by Vielma and Nemhauser (2011). This approach introduces additional continuous variables $\alpha_{s}\in {\left[0,1\right]}^{n_{p_{s}}+1}$ , $\beta_{s}\in {\left[0,1\right]}^{n_{q_{s}}+1}$ , $\gamma_{s}\in {\left[0,1\right]}^{\left(n_{p_{s}}+1\right)\times \left(n_{q_{s}}+1\right)}$ , and $n_{\delta }$ additional binary variables $\delta_{s}\in {\left\{0,1\right\}}^{n_{\delta }}$ , where $n_{\delta }=\left\lceil \log_{2}\left(\left(n_{p_{s}}+1\right)\times \left(n_{q_{s}}+1\right)\right)\right\rceil$ . The bilinear constraint $x\left[r_{s}\right]=x\left[p_{s}\right]x\left[q_{s}\right]$ is then reformulated into the following mixed-integer linear constraints:

$$\begin{array}{cc} & x\left[p_{s}\right]=\underset{i}{\sum }\alpha_{s}\left[i\right]x_{p_{s}}^{i},\;x\left[q_{s}\right]=\underset{i}{\sum }\beta_{s}\left[i\right]x_{q_{s}}^{i}\\ & x\left[r_{s}\right]=\underset{i}{\sum }\underset{j}{\sum }\gamma_{s}\left[i,j\right]x_{p_{s}}^{i}x_{q_{s}}^{j}\\ & \underset{j}{\sum }\gamma_{s}\left[i,j\right]=\alpha_{s}\left[i\right],\;\underset{i}{\sum }\gamma_{s}\left[i,j\right]=\beta_{s}\left[j\right]\\ & \alpha_{s},\beta_{s}\;\text{are in SOS}2\left(\text{enforced by}\;\delta_{s}\right) \end{array}$$ (3)

We refer readers to Vielma and Nemhauser (2011) for more details on SOS2 enforcement using binary variables. Briefly speaking, SOS2 constraint ensures that only two consecutive values in a vector can be non-zero, and these non-zero values must sum to 1. When applied to $\alpha_{s}$ , this constraint ensures that only two adjacent elements (e.g., $\alpha_{s}\left[i\right]$ and $\alpha_{s}\left[i+1\right]$ ) can be non-zero, effectively identifying the specific interval $\left[x_{p_{s}}^{i},x_{p_{s}}^{i+1}\right]$ where the variable $x\left[p_{s}\right]$ lies within. The exact pair of consecutive non-zero elements is determined by the binary variables $\delta_{s}$ .

For our data-driven approach, we use offline solved problems to train the learner. During online execution, the learner provides a complete set of binary variables that both fixes the binary variables in (1d) and identifies the appropriate intervals for $x\left[p_{s}\right]$ and $x\left[q_{s}\right]$ through predicting $\delta_{s}$ ’s, such that the bilinear constraints in (1e) are replaced with their linear approximations. This reformulation transforms the problem into a quadratic program (QP) that can be solved efficiently with fast convex optimization solvers. For the learning component, we have implemented the K-nearest neighbor approach proposed by Zhu and Martius (2020). The complete KNN-based algorithm is detailed in Algorithm 2.

Algorithm 2 MICP Online Solving Procedure.

Input: $\left\{\left(\Theta_{i},x_{i}\right)\right\}$ , Θ

1:  Find the K-nearest-neighbors $\Theta_{1}$ , …, $\Theta_{K}$ in $\left\{\Theta_{i}\right\}$ , sorted in order of increasing Euclidean distance $d\left(\Theta ,\Theta_{k}\right)$ . Retrieve $x_{1}$ , …, $x_{K}$ .
2:  **for** k=1,…,K do
3:   for s=1,…,S do
4:    Identify $\delta_{s}$ , through finding the interval $\left[x_{p_{s}}^{i},x_{p_{s}}^{i+1}\right]$ and $\left[x_{q_{s}}^{j},x_{q_{s}}^{j+1}\right]$ that $x_{k}\left[p_{s}\right]$ , $x_{k}\left[q_{s}\right]$ stays within. Replace (1e) with the linear constraints derived from (3) for the identified intervals, where the SOS2 constraints are satisfied by fixing the binary variables.
$$\begin{array}{ll} x\left[p_{s}\right]=\alpha_{s}\left[i\right]x_{p_{s}}^{i}+\alpha_{s}\left[i+1\right]x_{p_{s}}^{i+1},\; & x\left[q_{s}\right]=\beta_{s}\left[j\right]x_{q_{s}}^{j}+\beta_{s}\left[j+1\right]x_{q_{s}}^{j+1}\\ x\left[r_{s}\right]=\gamma_{s}\left[i,j\right]x_{p_{s}}^{i}x_{q_{s}}^{j}+\gamma_{s}\left[i+1,j\right]x_{p_{s}}^{i+1}x_{q_{s}}^{j} & \;+\gamma_{s}\left[i,j+1\right]x_{p_{s}}^{i}x_{q_{s}}^{j+1}+\gamma_{s}\left[i+1,j+1\right]x_{p_{s}}^{i+1}x_{q_{s}}^{j+1}\\ \gamma_{s}\left[i,j\right]+\gamma_{s}\left[i,j+1\right]=\alpha_{s}\left[i\right],\; & \gamma_{s}\left[i,j\right]+\gamma_{s}\left[i+1,j\right]=\beta_{s}\left[j\right]\\ \alpha_{s}\left[i\right]+\alpha_{s}\left[i+1\right]=1,\; & \beta_{s}\left[j\right]+\beta_{s}\left[j+1\right]=1\\ \alpha_{s}\left[i\right],\alpha_{s}\left[i+1\right],\beta_{s}\left[j\right],\beta_{s}\left[j+1\right],\gamma_{s}\left[i,j\right], & \gamma_{s}\left[i+1,j\right],\gamma_{s}\left[i,j+1\right],\gamma_{s}\left[i+1,j+1\right]\in \left[0,1\right] \end{array}$$
6:   Fix $x\left[j\right]=x_{k}\left[j\right],\;\forall \;j\in J$

7:   Solve the resultant QP using applicable solvers.
8:   if *successful*then
9:    return x

**10**:  return*nil*

### 3.2 Experimental problem formulations

In this section, we present two problems with two different robotic models that will serve as benchmarks for evaluating the data-driven MIBLP reformulation approaches described in Section 3.1. The first model is a linear inverted pendulum with soft contact walls. The second model is a single rigid body with mode switching and contact constraints. For each problem, we detail how they can be formulated as MIBLPs following the structure of problem (1).

#### 3.2.1 Linear inverted pendulum with soft contacts

For this problem, we model a linear inverted pendulum on a cart that can make contact with soft walls on either side. This example is a standard benchmark problem used to verify linear controllers with discrete contacts (Aydinoglu et al., 2023; Marcucci and Tedrake, 2020; Cauligi et al., 2021; Quirynen and Di Cairano, 2023).

The state vector is $x=\left[x_{c},\theta ,\;{\dot{x}}_{c},\;\dot{\theta }\right]$ , where $x_{c}$ is the cart position, ${\dot{x}}_{c}$ is the cart velocity, θ is the pendulum angle measured from the vertical upward direction, and $\dot{\theta }$ is the angular velocity. The control input u is the horizontal force applied to the cart.

We use the standard linearized dynamics for the inverted pendulum around its unstable equilibrium point (Marcucci and Tedrake, 2020). When the pendulum tip makes contact with walls on either side, contact forces are generated according to a soft-contact model. We implement this using two binary variables indicating contact with the left or right wall at a given time, respectively. The contact force is modeled as a linear spring with contact forces proportional to the penetration depth.

This problem introduces binary variables, but does not introduce bilinear constraints. Nevertheless, it still fits the MIBLP framework given by (1) and therefore serves as a valid benchmark problem here. In this formulation, the parameter Θ represents the initial state of the system, including cart position, velocity, pole orientation, and angular velocity.

#### 3.2.2 Single rigid body with mode switching and contacts

For this problem, we adopt the standard single rigid body dynamics formulation (Di Carlo et al., 2018), including translational and rotational dynamics. However, MIBLP formulation (1) allows arbitrary rotation angles and can model contact decisions as binary variables, avoiding the small angle approximation and pre-planned contacts used in Di Carlo et al. (2018). This enhanced formulation enables the planning of more aggressive and versatile motions, including transitions between quadrupedal and bipedal locomotion modes, as demonstrated in our experiments.

The continuous variables in this single rigid body formulation include position of geometric center of body p , velocity v , Z-Y-X Euler angles θ=[ϕ,θ,ψ] , angular velocity ω , contact positions $p_{i}$ and contact forces $f_{i}$ . One key aspect of MIBLP formulation is that it allows bilinear terms from the moment computation $p_{i}\times f_{i}$ , which represents the cross product between the moment arm and contact force for each toe. Another source of bilinear terms comes from the usage of full rotation matrix using Z-Y-X Euler angles [ϕ,θ,ψ] :

$$R=\left[\begin{array}{ccc} c\theta c\psi & s\phi s\theta c\psi -s\psi c\phi & s\phi s\psi +s\theta c\phi c\psi \\ s\psi c\theta & c\phi c\psi +s\phi s\theta s\psi & s\theta s\psi c\phi -s\phi c\psi \\ -s\theta & s\phi c\theta & c\phi c\theta \end{array}\right]$$ (4)

And the transformation from Euler angle rates to angular velocities ω :

$$\omega =\left[\begin{array}{ccc} c\theta c\psi & -s\psi & 0\\ c\theta s\psi & c\psi & 0\\ -s\theta & 0 & 1 \end{array}\right]\left[\begin{array}{c} \dot{\phi }\\ \dot{\theta }\\ \dot{\psi } \end{array}\right]$$ (5)

For the remaining dynamics equations, discretization using Euler forward integration, friction cone constraints, and leg workspace approximation as a box-shaped region, we follow the approach in Di Carlo et al. (2018).

For each bilinear term that appears in the dynamics, rotation matrix, and angular velocity computations, we augment the optimization variable x with a term that equals the multiplication of those variables (e.g., $c_{\theta \psi }=c\theta c\psi$ ). This single term replaces the bilinear expression, such that the equation is linearized. In the case where there is a trilinear term such as sϕsθcψ , we do this recursively by introducing two auxiliary variables: first one representing the bilinear term (e.g., $s_{\phi \theta }=s\phi s\theta$ ), turning the trilinear term into a bilinear one, then apply the same approach again. We apply standard piecewise linear approximations (Deits and Tedrake, 2014) to cθ and sθ via introducing additional binary variables. Through these techniques, constraints (4), (5), and the moment equation become linear and fit into the form of constraint (1b), while the bilinear equalities are captured in constraint (1e).

The robot is assumed to walk on flat terrain. For contact modeling, we introduce binary variables $c_{i}$ for each toe i , where $c_{i}=1$ indicates the toe is in contact with the ground (toe height = 0) and $c_{i}=0$ indicates the toe is in the air. When a toe is not in contact $\left(c_{i}=0\right)$ , its contact force is constrained to zero using:

$$|f_{i}|\leq f_{\max}c_{i}$$ (6)

On the other hand, when the leg is in contact $\left(c_{i}=1\right)$ , the z -height of its toe position is constrained to the ground:

$$p_{i,z}\leq M\left(1-c_{i}\right)$$ (7)

where M is a constant large enough such that when $c_{i}=0$ , the z-height of the toe is unconstrained.

To enable multi-modal locomotion, we define two distinct modes: quadrupedal and bipedal locomotion, represented by a binary variable $m_{k}\in \left\{0,1\right\}$ at each timestep k . In quadrupedal mode $\left(m_{k}=0\right)$ , all four legs follow an alternating contact pattern where legs lift to a predefined height $h_{l}$ and make periodic ground contact with the ground. In bipedal locomotion mode $\left(m_{k}=1\right)$ , the body is oriented with a pitch angle above a minimum threshold to maintain an upright posture, and the two front limbs are lifted above the predefined height and remain in the air, while only the two back legs alternate between contact and lift phases periodically.

These mode-dependent behaviors are enforced through the following constraints. For front legs (i∈{0,1}) , the contact sequence constraints are:

$$c_{i,k+1}\leq 1-c_{i,k}+m_{k}$$ (8a)

$$c_{i,k+1}\geq 1-c_{i,k}-m_{k}$$ (8b)

Which enforces periodic contact only in quadrupedal mode. For back legs (i∈{2,3}) , periodic contact constraints apply regardless of mode.

To enforce the upright orientation in bipedal mode, we add the constraint:

$$\theta_{k}\geq \theta_{\text{min}}-M\left(1-m_{k}\right)$$ (9)

where $\theta_{\text{min}}$ is the minimum pitch angle threshold and M is a large constant. This constraint is active only when $m_{k}=1$ (bipedal mode), enforcing $\theta_{k}\geq \theta_{\text{min}}$ .

The toe position constraints are:

$$p_{i,z,k}\leq h_{l}+Mm_{k}$$ (10a)

$$p_{i,z,k}\geq h_{l}-Mc_{i,k}-Mm_{k}$$ (10b)

These constraints implement the mode-dependent toe lifting behavior: in quadrupedal mode, legs alternate between ground contact ( $p_{i,z,k}=0$ when $c_{i,k}=1$ ) and lift height ( $p_{i,z,k}=h_{l}$ when $c_{i,k}=0$ ), while in bipedal mode, front legs are allowed to lift higher than the standard lift height.

The complete formulation incorporates standard single rigid body dynamics with rotational kinematics described by Equations 4, 5, and leg feasible kinematic region is approximated using box constraints, following (Di Carlo et al., 2018). Contact modeling constraints (Equations 6, 7) enforce the complementary relationship between contact forces and toe heights. Mode-dependent locomotion behaviors are captured through contact sequence constraints (Equation 8) for front legs, body orientation requirements (Equation 9) for bipedal mode, and toe position constraints (Equation 10) that implement mode-dependent prescribed lift heights. The objective function consists of multiple weighted terms to achieve stable locomotion and reach the target position. The primary goal is to minimize the squared distance between the body center and target position $\left(x_{\text{target}},z_{\text{target}}\right)$ throughout the trajectory with a term $\sum_{k=0}^{N}\|\left(p_{x,k},p_{z,k}\right)-\left(x_{\text{target}},z_{\text{target}}\right){\|}^{2}$ . To ensure smooth motion, we add penalizations on control effort (squared accelerations in x , z , and pitch angle θ ), body height variations between consecutive timesteps, and excessive foot movement during swing phases. Finally, to avoid unnecessary mode transitions between quadrupedal and bipedal configurations, we add a term $\sum_{k=0}^{N-1}{\left(m_{k+1}-m_{k}\right)}^{2}$ that penalizes mode switching.

The complete formulation results in an MIBLP of the form given in Equation 1. In this formulation, the parameter Θ represents the initial state of the system, including position, velocity, orientation, and angular velocity.

## 4 Results and discussion

### 4.1 Experiment on linear inverted pendulum with contacts

For our first benchmark problem, we implemented a linearized cart-pole system with soft contact walls on either side. The system consists of a cart with mass $m_{\mathrm{cart}}=1.0$ kg and an inverted pendulum with mass $m_{\mathrm{pole}}=0.4$ kg and length l=0.6 m. The system includes walls on both sides at distances $d_{\mathrm{left}}=d_{\mathrm{right}}=0.5$ m from the origin. When the pendulum tip contacts these walls, a soft contact force is generated proportional to the penetration depth, with stiffness coefficients $k_{1}=k_{2}=50$ N/m for the right and left walls, respectively. The control input is limited to |u|≤20 N, and the pendulum angle is constrained to |θ|≤π/2 . The control objective is to stabilize the pendulum in the upright position $x_{g}=\left[0,0,0,0\right]$ . We use a quadratic cost function with state cost matrix Q=diag(1,50,1,50) , control cost R=diag(1,0.1,0.1) , and terminal cost $Q_{\mathrm{ter}}$ computed from the Discrete Algebraic Riccati equation.

To build our dataset for data-driven acceleration, we created a dataset with 50 and 500 samples distributed across the state space. We generated solutions for a grid of initial conditions, varying cart position: [−0.3,0.3] m, pendulum angle: [−π/4,π/4] rad, cart velocity: [−0.5,0.5] m/s, and angular velocity: [−0.5,0.5] rad/s. For each set of initial conditions, we solved the complete MIBLP formulation using Gurobi (Gurobi Optimization, LLC, 2024) to obtain warm-start solutions.

For testing, we generated 200 random initial conditions different from the training set but drawn from the same distribution. These solutions were stored and used to warm-start both reformulation approaches. For the MPCC reformulation, we use IPOPT (Wachter, 2002) as the NLP solver for the complete formulation with complementary constraints. For the MICP reformulation, we use Gurobi as the convex optimization solver after fixing the binary variables.

**Results** Table 1 shows the computational performance comparison between different solving approaches. The “No Initial Guess MPCC” column represents solving the MPCC formulation with default zero initialization. The “Manual Initial Guess MPCC” column represents solving MPCC with a straight-line trajectory initialization that linearly interpolates all state variables from the initial state to the final upright equilibrium, with all control inputs and binary variables initialized to zero. The “Enhanced Manual Initial Guess MPCC” column represents solving MPCC with a physics-informed warm-start: if the pole is angling toward a wall, the trajectory assumes the pole tip will first move toward the wall at constant velocity, remain in contact for a specified duration, then move away from the wall at constant velocity; otherwise, it uses the straight-line interpolation. Our data-driven approaches included “Data-driven MPCC” using KNN to generate initial guesses for the MPCC formulation, and “Data-driven MICP” using KNN to predict binary variables for the MICP reformulation.

**TABLE 1:** Performance comparison of different solving methods for the cart-pole problem.

Metric	No initial MPCC	Manual initial MPCC	Enhanced initial MPCC	Data-driven (50)		Data-driven (500)	
				MPCC	MICP	MPCC	MICP
Amount of data	0	0	0	50	50	500	500
Success rate (%)^*^	0	42.5	58.0	63/74/76	43/54/56	78/88/90	55/63/71
Avg. solve time (ms)	N/A	22.0	22.0	25.0	0.6	25.0	0.6
Avg. # of neighbors	N/A	N/A	N/A	1.28	1.30	1.26	1.28
Avg. objective value	N/A	250.0	250.0	978.49	1,358.15	741.68	1,518.59
Solver	IPOPT	IPOPT	IPOPT	IPOPT	Gurobi	IPOPT	Gurobi

*Success rates shown for K = 1/2/3 KNN, neighbors for data-driven methods.

We evaluated the success rate, solving time, and solution quality for each method. The “Amount of data” row indicates the number of offline-solved problem instances in the dataset (zero for non-data-driven methods). The “Success rate” row shows the percentage of test problems solved successfully: for manual initialization methods, this represents a single solving attempt; for data-driven methods, the format a/b/c indicates cumulative success rates when trying K=1/2/3 nearest neighbors sequentially until a solution is found. The “Average solve time” represents the mean computational time across successful problem instances, including all KNN neighbor attempts until success. We also provide a histogram showing the distribution of solving times in subplot (A) of Figure 1, indicating how many problem instances are solved within each time interval for data-driven MPCC with 50 training samples. The distribution for 500 training samples exhibits a similar pattern. For data-driven MICP, all problem instances are solved within 1 m. The “Avg. # of neighbors” indicates the average number of KNN neighbors attempted before finding a successful solution. The remaining rows show the average objective value among successful solutions and the solver used.

**Analysis** First, MPCC with default zero initialization completely fails to find feasible solutions (0% success rate), while manual straight-line initialization achieves moderate success (42.5%). The enhanced manual initialization achieves a higher success rate of 58.0%. However, designing such heuristics requires problem-specific insight and manual effort that may not scale to more complex robotic systems with higher-dimensional state spaces. The data-driven MPCC approach significantly improves performance, achieving a 76% success rate with just 50 data samples. The data-driven MICP formulation achieves a lower success rate (56%) with the same amount of data. When increasing the dataset to 500 samples, both approaches improve further, with MPCC reaching a 90% success rate and MICP improving to 71.0%. The MICP approach offers nearly a 100 × speedup with 50 samples and 45 × with 500 samples, partly due to the commercial Gurobi solver compared to the open-source IPOPT solver used for the MPCC approach.

It is important to note that the relaxation parameter ϵ plays a crucial role in the MPCC formulation to trade off success rate with numerical accuracy. In this experiment, we selected ϵ=0.005 for a good balance. All the resultant binary variables are within 0.01 of their true binary values, and the success rate is reasonable. If we were to reduce this value to ϵ=0.001 , the accuracy of binary solutions is higher at the cost of decreasing the success rate.

### 4.2 Experiment on single rigid body with contacts

For this experiment, we evaluate our MIBLP motion planner on a quadrupedal robot model. We use the Spine-enhanced Climbing Autonomous Legged Exploration Robot (SCALER) for demonstration, and implement an SRB model that matches the robot’s physical properties. The total mass is set to 10.0 kg , the moment of inertia is [0.15,0.30,0.35] $kg\cdot m^{2}$ , and the body dimensions are 0.5 m×0.3m×0.2m . For detailed descriptions of this platform, we refer readers to Tanaka et al. (2022). The SCALER robot is equipped with 6-axis force-torque sensors at each limb, allowing us to implement admittance control that enables tracking of the planned contact forces (Schperberg et al., 2023).

**FIGURE 2:**
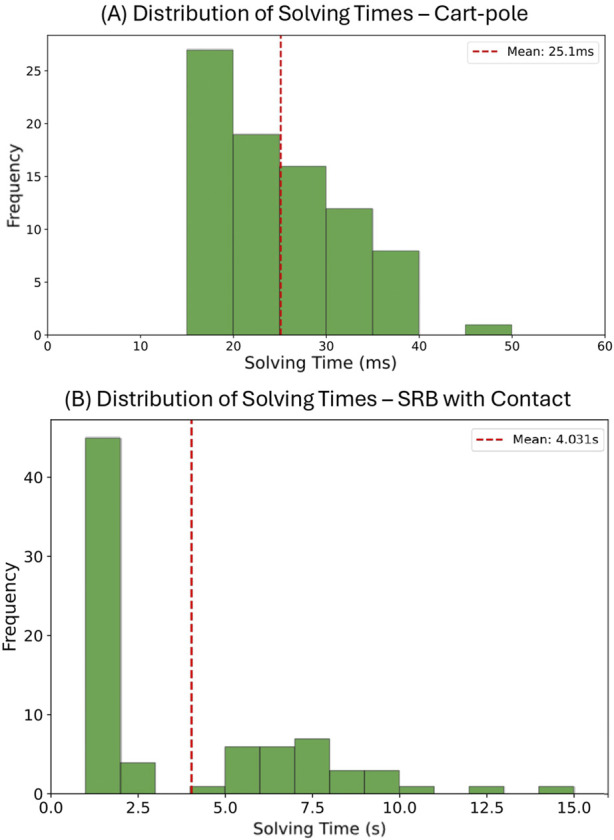
Solving time distribution for data-driven MPCC. **(A)** Cart-pole with 50 samples. **(B)** SRB with contacts using 30 samples.

To showcase the advantages of our MIBLP formulation, which can plan trajectories without pre-assigned gait sequences or small angle approximations, we design a challenging scenario. The environment contains a low-clearance obstacle positioned at height $h_{\text{obs}}$ between x=1.5m and x=2.5m that requires the robot to pass underneath. The target is an elevated platform located at height $h_{\text{target}}>h_{\text{obs}}$ , requiring the robot to reach upward to achieve the goal position. The robot begins in bipedal configuration with an upright posture, then transition into quadrupedal mode to crawl beneath the low-clearance obstacle, and finally return to bipedal configuration to reach the elevated target position. The scenario is shown in Figure 2.

We sampled 30 and 80 initial conditions with horizontal positions spanning a 1-m range and initial orientation varying within [−π/6,π/6] from the vertical orientation. We solved these problems using a two-stage approach: first, we solve a low-accuracy MICP formulation using coarser McCormick envelopes indicated in Table 2 (column “data collection”, $p_{\text{sh},i}$ denotes the shoulder position for leg i relative to the center of mass) to generate initial solutions, then refine them through the MPCC formulation using the MICP solution as a warm-start to ensure bilinear constraints are exactly satisfied. This approach can be viewed as a simplified single-iteration case of iterative schemes such as Alternating Direction Method of Multipliers (ADMM) and Generalized Benders Decomposition (GBD). For applications requiring higher accuracy or when the initial MICP solution fails to converge in the MPCC refinement step, these full iterative schemes can be utilized.

**TABLE 2:** Segmentations of the nonconvex variables.

Variable	Range	# Intervals data collection	# Intervals testing
θ (rad)	[−π/2,π/2]	4	5
$p_{i}-p$ (m)	$p_{\text{sh},i}\pm 0.16$	2	3
$f_{i}$ (N)	[−150,150]	2	4

For testing, we generated 100 random initial conditions that were different from the training dataset and used them to evaluate both the MPCC and MICP formulations. Higher accuracy McCormick envelopes are used in these MICP tests to ensure accurate approximation of the bilinear constraints, with the number of segmentations given in Table 2 (column “testing”).

**Results** Table 3 shows the computational performances of different solving methods. “Manual Initial Guess MPCC” uses a common straight-line trajectory to connect the initial and goal configurations as the initial guess for the MPCC solver. All binary variables are initialized to 0.5 to provide a neutral starting point without biasing toward any particular contact mode. “Two-stage MICP-MPCC” refers to the two-stage approach used for data collection described previously: first solving a coarse MICP formulation, then refining through MPCC with the MICP solution as warm-start. “Data-driven MPCC” uses our KNN approach to generate initial guesses for the MPCC formulation, while “Data-driven MICP” uses KNN to predict binary variables for the MICP reformulation.

**TABLE 3:** Performance comparison of different solving methods for SRB with mode transitions and contacts.

Metric	Manual initial	Two-stage	Data-driven (30)		Data-driven (80)	
	Guess MPCC	MICP-MPCC	MPCC	MICP	MPCC	MICP
Amount of data	0	0	30	30	80	80
# of KNN neighbors (K)	N/A	N/A	2	3	2	3
Success rate (%)^*^	0.0	N/A	58/84	5/16/29	50/86	21/35/47
Avg. solve time (s)	N/A	13.25	8.13	0.19	6.91	0.19
Avg. # of neighbors	N/A	N/A	1.32	1.72	1.32	1.91
Avg. objective value	N/A	598	622	608	629	608
Solver	IPOPT	Gurobi + IPOPT	IPOPT	Gurobi	IPOPT	Gurobi

*Success rates shown for K = 1/2 (MPCC) or K = 1/2/3 (MICP) KNN neighbors.

**Analysis** We observe that both data-driven approaches benefit substantially from warm-starting with the collected dataset. Manual initialization achieves a 0% success rate, failing to find feasible solutions for any test cases. The two-stage MICP-MPCC approach used for data collection demonstrates feasibility but requires 13.25s on average to solve. In contrast, with 30 training samples, MPCC achieves up to 84% success rate while MICP reaches 29%; with 80 training samples, MPCC improves to 86% while MICP achieves 47%.

Given the same amount of data, MPCC shows higher success rates, matching the trend observed in the cart-pole experiment where MPCC also outperformed MICP with limited training data. For computational speed, MICP demonstrates a significant advantage, solving problems in approximately 0.19s compared to 6.91−8.13s for MPCC, more than 35 × faster. This speedup makes MICP particularly suitable for real-time applications.

### 4.3 Discussion on experiment results

According to our experiments on both the linear inverted pendulum and single rigid body control problems, data-driven methods significantly improve the performance of both MPCC and MICP reformulations. As we increased the amount of data, we observed a general trend of improvement in success rates for both approaches. The key distinction between MPCC and MICP reformulations lies in the solving speeds and the amount of data used to achieve similar success rates.

For both the linear inverted pendulum problem and the single rigid body problem, the MPCC formulation achieves higher success rates than the MICP formulation with the same amount of data. We noticed that IPOPT rarely modifies the initial binary guesses for x[j],j∈J . Therefore, this difference stems from how the bilinear constraints (1e) are handled. The MICP reformulation converts them into multiple linear envelopes with additional binary variables $\delta_{s}$ . The learner directly provides $\delta_{s}$ , which fixes the intervals $\left[x_{p_{s}}^{i},x_{p_{s}}^{i+1}\right]$ and $\left[x_{q_{s}}^{j},x_{q_{s}}^{j+1}\right]$ within which $x\left[p_{s}\right]$ and $x\left[q_{s}\right]$ must lie. The convex solver then finds $x\left[p_{s}\right]$ and $x\left[q_{s}\right]$ inside these intervals. If the learner predicts incorrect intervals, the resulting problem becomes infeasible, requiring the learner to try alternative neighbors. Thus, a sufficiently large dataset is necessary to train the learner to predict the correct intervals consistently. In contrast, for MPCC re-formulation, KNN provides initial guesses for $x\left[p_{s}\right]$ , $x\left[q_{s}\right]$ , then the NLP solver uses numerical schemes to satisfy (1e) and other constraints. Since NLP solvers are designed to satisfy nonlinear constraints, even restoring from infeasible initial guesses, the MPCC approach demonstrates greater robustness with limited data, resulting in higher success rates.

On the other hand, the MICP formulation demonstrates significantly faster solving speeds when provided with sufficient data. This advantage likely stems from the efficiency of modern convex optimization solvers. For MPCC problems solved with IPOPT, even when we relax the complementarity constraint (2) to values between 0.005 and 0.01 and provide quality warm-start solutions, the solver frequently encounters numerical difficulties and shows slow convergence. The complementarity constraint (2) remains inherently challenging to satisfy. Notably, for the linear inverted pendulum with contact, directly solving using MICP with Gurobi can achieve global optimality at 300 Hz without any data-driven acceleration (Lin, 2024). In addition, numerical accuracy remains a concern with the MPCC approach. Although we only relax constraint (2) to values between 0.005 and 0.01, less than 1% of the binary values, these small relaxations can still lead to accuracy issues in certain cases.

The amount of training data required for effective data-driven MIBLP acceleration depends on the dimensionality of Θ and the problem complexity, such as the number of constraints. Previous theoretical work by Hauser (2016) has established worst-case bounds for data requirements in parametric optimization problems, showing that the amount of data needed scales exponentially with the dimensionality of Θ . While Hauser (2016) assume globally optimal solutions, they provide useful guidance for understanding scaling behavior when using locally optimal warm-start solutions. In practice, the data requirements can be significantly better than these theoretical bounds, especially for the MPCC formulation since NLP solvers are often capable of converging from poor initial guesses to feasible solutions.

Therefore, despite the benefits of data-driven acceleration for solving optimization problems, different formulations exhibit distinct performance characteristics and dataset requirements. The choice of problem formulation is crucial for maximizing the effectiveness of data-driven optimization methods.

## 5 Conclusion

In this paper, we present a comparative study of different reformulation strategies for solving MIBLPs aided by data for robot motion planning applications. We conducted numerical experiments on two motion planning problems for a linear inverted pendulum with contact walls and for quadrupedal locomotion involving quadruped-bipedal mode transformations.

While this work focuses on reformulating the MIBLP problem into more tractable problem types such that existing methods can directly apply, future work can focus on developing innovative data-driven methods tailored specifically to MIBLPs.

## Data Availability

The original contributions presented in the study are included in the article/supplementary material, further inquiries can be directed to the corresponding author.
